# Evaluating referrals between rural district hospitals and a regional hospital in South Africa

**DOI:** 10.4102/phcfm.v17i1.4956

**Published:** 2025-07-09

**Authors:** Kambola D. Ngoie, Louis Jenkins, Johann Schoevers

**Affiliations:** 1 Department of Family and Emergency Medicine, Faculty of Medicine and Health Sciences, Stellenbosch University, Cape Town, South Africa; 2 Primary Health Care Directorate, Family, Community and Emergency Care, Faculty of Medicine and Health Sciences, University of Cape Town, Cape Town, South Africa; 3 Department of Family and Emergency Medicine, George Hospital, Western Cape Department of Health, George, South Africa

**Keywords:** referrals, district hospitals, regional hospital, rural districts, South Africa

## Abstract

**Background:**

Efficient referral systems are essential for improving healthcare and patient outcomes, especially in resource-limited settings where access to public specialist care is limited by too few specialists, growing populations and constrained resources impacting non-emergency and emergency referrals. District hospitals (DHs) must ensure that patients receive the appropriate level of care. High-quality referral systems are necessary for the cost-effective flow of patients between district and regional hospitals (RHs).

**Aim:**

This study aimed to evaluate emergency and non-emergency patient referral processes between DHs and the RH in two districts in South Africa.

**Setting:**

Ten DHs and the RH in the Garden Route and Central Karoo districts in South Africa.

**Methods:**

A mixed-methods design incorporated quantitative survey data and qualitative thematic analysis to provide a comprehensive understanding of referral processes. The study population included all doctors working at 10 DHs and the RH, with 120 voluntary participants.

**Results:**

Key findings revealed disparities in referral satisfaction between emergency (66%) and non-emergency (59%) referrals. Communication breakdowns and systemic barriers hindered timely access to specialist care, mismatched expectations and understanding, coupled with inconsistent referral guidelines. Inadequate capacity building increased inappropriate referrals.

**Conclusion:**

Communication breakdowns and differing expectations between DHs and the RH regarding available resources and services negatively impacted referrals. Improved communication, targeted outreach, capacity-building initiatives, stronger collaborative relationships and standardisation of processes could enhance patient referral efficiency.

**Contribution:**

This work adds new knowledge to patient referrals between rural district and regional hospitals in resource-limited contexts, highlighting the complexity of the referral process.

## Introduction

Patient referrals follow a structured sequence of health-related steps beginning with the patient assessment, progressing to formal referral, leading to entry into the designated care setting and concluding with completion of the referral.^1^ It entails clear communication and coordination between referring and receiving doctors to ensure efficient use of resources and high-quality care.^2^ Effective referrals are essential for achieving high standards of healthcare, ensuring continuity of care, optimising good utilisation of resources and improving patient outcomes.^3^

The health system in South Africa (SA) has private and public sectors.^4^ Over three-quarters of the population utilises the public health service, a system built on the primary health care (PHC) model recommended by the World Health Organisation (WHO).^5^ There is a hierarchical relationship between the levels of care in the public health system. Community health centres (CHCs) and clinics refer patients to district hospitals (DHs); DHs refer to the regional hospital (RH) and RHs refer to the tertiary hospital (TH) and backwards.^4^ The public health referral system encounters challenges including: (1) inappropriate self-referral to hospitals, (2) lack of feedback between health care providers, (3) unclear and ill-defined linkages between health facilities and (4) limited resources and ineffective organisation.^4,6,7^

District hospitals support PHC and provide generalist services, ensuring that patients get the appropriate level of care and continuity of care.^4,8^ Patients may receive specialist care at DHs through outreach programmes. In most cases, patients are referred to a higher care facility and require transit across the primary–secondary care interface.^9^ The reasons for a higher level of care referral may be divided into two groups: non-emergencies and emergencies, which might include referral for diagnosis, lack of resources, investigations, seeking management advice and seeking treatment above the scope of PHC. Early referral for patients with non-emergency or emergency conditions is advisable when the prognosis is favourable.^10,11^

Coordination between primary and secondary care relies on the PHC provider’s ability to navigate and connect different levels of care.^9,11,12^ For example, to improve the coordination of services in the Western Cape (WC), the provincial health department has organised the health system into five geographic areas, each with a RH receiving referrals from specific DHs, which facilitate access to speciality care for the population in that geographic area.^13^

Access to higher levels of care is becoming increasingly difficult because of the limited number of specialists in the public health sector, the increasing demand for specialist care in society and the limited resources available.^7^ It is imperative to have a high-quality referral system to maintain an efficient, patient-centred and cost-effective flow through the primary–secondary care interface.^10^ Referrals should be appropriate and well-communicated to ensure adequate and timely care.^7,13^ There are three essential aspects to consider in determining the quality of the referral process: (1) Establish whether the referral is required (necessity), (2) identify the level of care needed for the patient’s condition (destination) and (3) identify how the patient will get to the destination (quality).^14,15^

Referrals from primary care to DHs were shown to be ineffective, with suggestions that regular outreach to PHC facilities, patients and healthcare providers, education on the referral system, medication availability at PHC and increased clinic working hours could improve referrals.^8^ Another study of transfers from CHCs to secondary hospitals of patients requiring emergency treatment revealed that resource allocation and training at the CHCs could have prevented more than one-fifth of transfers to the higher level.^16^

Other studies identified various system deficiencies at the primary–secondary interface: (1) Clinical decision-making and referral errors, (2) level management of flows between primary and secondary care, (3) communication systems, and (4) monitoring the quality of care.^1,10^ These deficiencies were linked to different interventions, including standardised referral processes with supporting guidelines, e-referrals, care pathway management, regular measurement of the referral process in terms of initiation of referral, accessibility to specialist care, coordination of primary–secondary care and quality (timeliness and satisfaction).^10^

Recent research has focused on electronic means to facilitate referrals, improve patients’ access to speciality care, increase support of PHC and prevent incomplete and inappropriate referrals.^1,17,18^ Using a consultation application and smartphone referral in areas with limited resources facilitated accessibility to specialists, improved patient care at the referring hospital and supported adequate patient management before the transfer.^6,18^

Existing research often focused on high-income settings or single-faceted issues such as communication, e-referrals and timeliness. Limited evidence exists on multi-dimensional aspects of patient referrals in resource-constrained districts in South Africa. This study aimed to address this gap by providing a comprehensive evaluation of the referral processes for non-emergency and emergency conditions between DHs and the RH in South Africa’s Garden Route (GR) and Central Karoo (CK) districts. The objectives included: (1) to describe referral practices for emergency and non-emergency conditions, including referral pathways for subspeciality care; (2) to determine the perceived quality of referrals, including different modes of communication; (3) to determine doctors’ satisfaction with the referral processes; and (4) to understand the challenges of the current referral processes.

## Research methods and design

### Study design and setting

This was a convergent mixed methods design utilising a cross-sectional survey. Online questionnaires were used to collect quantitative data and free text responses.

The setting was the WC province of SA, situated in the southwest with over 7.4 million population.^19^ The province has six districts and the study focused on the GR and CK districts. The GR district has six DHs and 28 public ambulances serving 838 457 people (0.4 emergency medical services [EMS] per 10 000).^20^ The CK is located north of the GR, with an estimated population of 102 173 served by four DHs and 16 ambulances (2.1 EMS per 10 000 people).^21^ The EMS transports patients with emergency conditions using ambulances, while Healthnet services transport patients with non-emergency conditions. The CK and GR districts are part of one geographic health area called the Rural East Cluster. Patients in this geographic area are referred to the George Regional Hospital (GRH) in the city of George. These two districts and the WC province have an increased number of non-communicable diseases and injuries but a decrease in infectious diseases.^22^

Referrals are divided into two pathways: Non-emergency patients are referred to specialist departments mainly through email but sometimes via phone calls, face-to-face conversations, the Vula® app or WhatsApp®. Specialists’ opinions are provided through the same channels. These patients are seen by the specialist at their respective DH during outreach or at the RH outpatient department, for which transport then needs to be organised. Emergency patient referrals are done mostly via phone calls to discuss patients or get advice. In addition, some departments require smartphone communication apps (WhatsApp communication or the Vula^©^ app), which is an e-referral app providing secure medical communication for referrals.^23^ Patients who are accepted for transfer are sent to the RH via the EMS.

The study included 10 DHs and one RH. While DHs in this area support PHC, provide generalist care and ensure continuity of care, GRH supports DHs with speciality services through its specialist outreach programme and by receiving referrals. As the only RH (266 beds) in the area, it provides district (Family Medicine), regional (Surgery, Internal Medicine, Paediatrics, Obstetrics and Gynaecology, Psychiatry and Orthopaedics) and some tertiary services, including Neurosurgery, Urology, Ear, Nose and Throat, Ophthalmology, Oncology and Neonatology. In addition, the hospital has the only computerised tomography scanner, high care unit (which functions as an ICU) and renal dialysis unit in this geographic area.

### Study population and sampling

The study population included all doctors working in the 10 DHs and the RH. This included medical managers, clinical managers, family physicians, family medicine registrars, medical officers, community service doctors and interns from the DH, and the medical manager, Heads of clinical units (HOCUs), specialists, registrars, medical officers and community service doctors from the RH. The inclusion criteria were all doctors working at DHs and the RH. Exclusion criteria were locum doctors working at DHs and the RH and interns at the RH, as they typically did not receive referrals. According to the district doctors list (published April 2024), GR and CK DHs had 109 doctors, 77% working in the GR and 23% in the CK. George Regional Hospital had 150 doctors, including 39 interns, 11 community service doctors, sessional and full-time medical officers, registrars, consultants and HOCUs. No sampling occurred, and the study invitation and questionnaires were sent to all potential participants.

### Study questionnaire

The researcher developed two draft questionnaires in English (one for DH participants and one for the RH participants) after reviewing the literature and conducting interviews with senior DH doctors (with over 10 years of experience) to gain a broad understanding of referral-related issues. Face validity was sought through multiple reviews and discussions with the supervisor and co-supervisor, both family physicians with research experience. Subsequently, the questionnaires were finalised and entered into REDCap^©^ (a secure Web-based software platform designed to support electronic data capture for research studies) hosted at the University of Stellenbosch.^24,25^ The questionnaires were piloted by five family physicians at both DHs and RH for content validation. Following validation and piloting, final revisions were made, and the researcher generated the final REDCap questionnaires and links for distribution to participants via email. The final questionnaires incorporated Likert scales and free text formats organised in sections linked to the objectives. (see Online Appendix 1 and Online Appendix 2)

### Data collection and analysis

District hospital managers and RH HOCUs facilitated the dissemination of the survey links to all potential participants according to the study inclusion criteria within their respective institutions and departments using their preferred communication channels. Reminders (email and telephonic) by the principal researcher to the institutions encouraged participation during the data collection period from 31 March 2024 to 02 July 2024. Voluntary, informed consent was sought from potential participants.

Data were collected using REDCap^©^ and extracted from the platform as a CVS file, cleaned, reviewed and screened for incompleteness and duplication. Incomplete data were excluded from the analysis. Quantitative data were analysed using Stata Statistical Software^©^ to calculate simple descriptive statistics including frequencies, means and medians depending on the distribution of the data, with the help of the Stellenbosch University Biostatistics Department.

Free text responses were extracted from REDCap, placed in a separate file and shared with the two study supervisors. Qualitative responses were analysed using the Braun & Clarke six-phase guide for thematic analysis.^26^ The steps include that the researcher and supervisors reviewed the free text responses multiple times for familiarisation, grouped the data and deductively generated initial codes, which were then organised into themes.

### Reflexive positioning of the researcher

The researcher is an experienced senior doctor training as a family medicine registrar. He has been working in the Mossel Bay subdistrict in the GR district for the last 3 years. He is quite familiar with many of the doctors at the RH and a smaller number of doctors at some of the other DHs. To minimise possible bias as a result of this familiarity, he had many reflexive conversations with his supervisors and stayed aware of his position as a DH family medicine registrar.

### Ethical considerations

Ethical clearance to conduct this study was obtained from the Stellenbosch University Human Research Ethics Committee (No. S23/07/164) and institutional approvals from the Western Cape Government, Provincial Health Research and Evaluation department (No. WC_202401_033).

## Results

There were 120 respondents, of which 109 completed the survey questionnaires. Sixty-eight (62%) were from DHs and represented different positions, while 41 (38%) were from various departments and positions at the RH. Overall characteristics were similarly distributed between the DHs and RH, except that RH respondents were more likely to have more years of service than DH participants (*p* 0.019). Free text responses were provided by 63% of respondents (*n* = 82), representing both DHs (*n* = 68, 83%) and RH (*n* = 14, 17%) (see Table 1).

**TABLE 1:** Characteristics of study participants (*N* = 120).

Characteristics	District hospitals		Regional hospital	
	*n*	%	*n*	%
**Total number of participants**	75	100	45	100
Completed	68	91	41	91
Not completed	7	9	4	9
Free text respondents	68	91	14	30
**Place of work**				
Mossel Bay Hospital	18	26	-	-
Knysna Hospital	21	31	-	-
Oudtshoorn Hospital	7	10	-	-
Riversdale Hospital	6	9	-	-
Beaufort West Hospital	8	12	-	-
Uniondale Hospital	2	3	-	-
Ladysmith Hospital	1	2	-	-
Murraysburg Hospital	2	3	-	-
Prince Albert Hospital	0	0	-	-
Laingsburg Hospital	3	4	-	-
**Family Medicine (including ENT, Urology and Dermatology) and Emergency Medicine**	-	-	11	27
Surgery	-	-	6	15
Internal medicine	-	-	5	12
Paediatrics (including neonatology)	-	-	2	5
Obstetrics and gynaecology	-	-	10	24
Orthopaedics	-	-	2	5
Psychiatry	-	-	2	5
Radiology	-	-	1	2
Ophthalmology	-	-	1	2
Oncology	-	-	1	3
**Ranking**				
Interns	9	13	-	-
Community service doctor	20	30	1	3
Medical officer	22	32	25	61
Registrar	5	7	2	5
Clinical manager	2	3	-	-
Medical manager	2	3	1	2
Family physician	8	12	2	5
Other consultant	-	-	7	15
HOCUs	-	-	3	7
**Length of service**				
< 6 months	24	35	4	10
6 months to 1 year	7	10	3	7
1 year to 3 years	7	11	6	15
> 3 years	30	44	28	68
**Previous district hospital work experience**				
Yes	-	-	30	73
No	-	-	11	27
**Referral guidelines awareness**				
Yes	46	68	25	61
No	12	18	5	12
Not sure	10	15	11	27

HOCU, heads of clinical unit; ENT, ear, nose, throat.

Participants expressed a difference between satisfaction levels for emergency and non-emergency referrals. Emergency referrals had higher satisfaction (66%) and a stronger belief in improved patient outcomes (73%) compared to non-emergency referrals (59%) satisfaction, with 51% believed it improved outcomes. District hospital respondents generally described non-emergency referrals to the RH as more challenging than emergency referrals. While the majority (71%) described non-emergency access as ‘accessible with some difficulties’, 24% found it ‘difficult to access’. In contrast, emergency referrals were perceived as more accessible, with most respondents finding them either ‘accessible with some difficulties’ (62%) or ‘easily accessible’ (32%). Referrals back from the RH to DHs were considered ‘easily accessible’, indicating a smooth transition for patients returning to local care.

Regarding referrals of patients needing sub-speciality care, participants’ views on the referral processes revealed a preference for direct referrals from the DH to a TH in some cases, bypassing the RH. Most respondents from both DHs (73%) and RH (54%) supported direct referrals for selected patients. This preference was often driven by existing RH advice to refer directly some new or follow-up patients with tertiary care needs:

‘Tertiary hospital specialised departments referrals to our department from DHs that result in the consultants at GRH doing secretarial work and forwarding referrals onto TH.’ (SP, P72, RH)

Regarding direct DH referrals to THs, only 34% of DH participants perceived this to have an improvement in the quality of patient care. Most DH participants (60%) supported the necessity of RH involvement in all tertiary referrals. The frequency of direct referrals from DHs varied considerably from less than two to more than five patients per DH doctor per year, with 40% of respondents having referred between two and five patients per year.

The qualitative analysis revealed six major themes: (1) Standardised and accessible referral guidelines, (2) active outreach and capacity building, (3) standardised communication, (4) systemic barriers hindering timely access to care, (5) mismatched expectations and understanding, and (6) building relationships (see Table 2).

**TABLE 2:** Themes from free text responses.

Themes	Participants group
1. Standardised and accessible referral guidelines	Both DHs and RH but mostly juniors from DHs
2. Active outreach and capacity building	Opinions expressed by both DHs and RH seniors and juniors
3. Standardised communication	DHs juniors and seniors
4. Systemic barriers hindering timely access to care	DHs views, both juniors and seniors
5. Mismatched expectations and understanding	Both DH’s and RH’s opinions
6. Building relationships	Views noted from both RH and DHs, juniors and seniors

DH, district hospital; RH, regional hospital.

### Standardised and accessible referral guidelines

Awareness of referral guidelines was relatively similar between the RH and DHs. Many DHs and RH participants (35%) remained unsure or uninformed about the existence of guidelines. Among those who were aware (60%), a lack of clarity regarding contents (referral criteria and pathways) raised concerns about the effectiveness of guidelines dissemination and usefulness.

Echoing the quantitative findings, participants emphasised the need for improved referral guidelines. Participants expressed a strong desire for consistency in referral processes and suggested a single, unified, simple and easily accessible referral guideline to minimise confusion arising from department-specific variations:

‘A standardised referral pathway for all speciality departments. Shared from one source to all DHs. Not separate requests for referral processes from each department.’ (CM, P6, DH)

This was underscored by the need for clear, accessible, unambiguous and consistently updated comprehensive referral guidelines that clearly outlined the boundaries between DH and RH care:

‘Clear distinct guidelines are needed to reduce the grey zone …RH protocols could be better distributed so that treatment that can be given at DHs level can be administered, whilst also ensuring a clear cut-off point for referral.’ (MO, P29, DH)

‘Give clear guidelines (typed out) that are sent out to each hospital annually … compile one document summarising referral pathways for all departments. This will help all new doctors … as well as keeping the rest of team up to date on what is expected of us from each department.’ (FM Reg, P60, DH)

### Active outreach and capacity building

Respondents highlighted the potential of outreach programmes to extend beyond direct patient care and to serve as valuable opportunities for teaching and capacity building. They envisioned these programmes as platforms for skills transfer, training sessions, protocol updates and fostering a collaborative environment for sharing best practices and discussing expectations. Participants believed that increased interaction and knowledge exchange through structured outreach programmes would encourage a greater understanding of each other’s contexts, leading to improved communication, stronger relationships and, ultimately, enhanced patient care across the healthcare system:

‘Skills transfer at outreach clinics, training sessions, regular M&M, updated protocols, better note keeping, accurate communication of clinical fact and feedback.’ (MO, P91, RH)

‘Tutorial teaching during outreaches that all the doctors can attend, not just the one doctor who’s assigned to help with outreach clinic.’ (CS, P40, DH)

‘Outreaches to meet each other face to face. A better understanding of what happens at both district and regional hospitals by both parties is essential.’ (MO, P39, DH)

Participants from DHs and the RHs expressed a strong need for a two-way learning process, with suggestions for RH doctors to spend time at DHs to gain a deeper understanding of their resources and challenges and for DH doctors to spend time at the RH to gain an understanding of RH resources and workflows:

‘In-reach doctors joining us for a short period in the unit.’ (SPC, P80, RH)

‘Rotation of regional department medical officers or registrars in district facilities and health systems.’ (FM Reg, P24, DH)

### Standardised communication

Participants’ responses on the current mode of communication revealed that phone calls (98%) were mostly used for emergencies, while non-emergency communication was more fragmented, with emails (72%) being the most common. The perceived effectiveness of these communication modes was mixed. While emails for non-emergency referrals were deemed ‘fair’ by 57%, 56% rated phone calls ‘good’ for emergencies, and 30% found them as only ‘fair’. This suggested that even the dominant mode of communication had challenges.

Most participants (76%) expressed a desire for a single mode of communication from DHs to all RH departments. Preferences varied, with the electronic app (Vula) being a favoured option (52%) followed by email (37%) for non-emergency referrals, while phone calls (69%) were preferred over the electronic app (Vula) for emergency referrals:

‘Universal discussion pathways in the district’ (FP, P38, DH/CS, P 59, DH)

‘[*I*]nstitute Vula referrals as standard for all referrals across the district.’ (CM, P27, DH)

While praised for its functionality, the electronic app (Vula) faced challenges because of inconsistent adoption across RH departments:

‘Vula is great! The problem is individuals in specific departments who refuse to use it.’ (FP, P28, DH)

This desire for standardisation extended to establishing clear procedures for communication, particularly in emergencies:

‘It would be very beneficial if, in cases of true emergencies, Vula can be bypassed.’ (MO, P 50, DH)

Participants were concerned about inconsistency in the adoption of technology like the Vula app across departments, creating communication delays, particularly in urgent situations where there was a preference for direct communication using phone calls:

‘Quick response times are needed, Vula is not well established for all departments. Phone calls are ideal.’ (CM, P6, DH)

Feedback practices emerged as another area requiring attention. Participants indicated a lack of emphasis on feedback from both sides. A substantial proportion of DHs reported never receiving feedback on their referrals (non-emergency 32%, emergency 35%), and even when provided, feedback was often rated as merely ‘fair’ (51% of DHs for non-emergency and emergency referrals). District hospital respondents themselves infrequently requested feedback, with only 27% doing so. This could potentially be linked to many factors, including a lack of awareness about the importance of feedback, not getting used to giving or receiving feedback or a lack of effective feedback communication.

### Systemic barriers hindering timely access to care

Challenges in the referral process impacted both non-emergency and emergency referrals. One common concern included communication barriers experienced by DHs caused by long response times for non-emergency referrals, with 32% of referral responses perceived to take a full week and some ranging from less than a week to over a month, creating uncertainty for both patients and referring physicians. Furthermore, negative attitudes from the RH staff during referral exacerbated the challenges faced by DHs. This perception was evident in the experiences shared by DH participants:

‘Not getting the response immediately and having to arrange an appointment with the patient.’ (MO, P39, DH)

‘Some departments are difficult.’ (CS, P66, DH)

These experiences underscored a need for improved communication and a more collaborative and respectful approach between DHs and RHs. As some participants suggested:

‘Improved attitude, respect and friendliness go a long way.’ (MO, P35, DH)

‘Behaviour change at DH and RH, seeing both sides of the story.’ (FP, P23, DH)

Less frequently perceived challenges included quality of advice, availability of call rosters and switchboard issues:

‘Sometimes unsure who to discuss with.’ (CS, P58, DH)

‘Not knowing who to contact on a specific day [*or*] time.’ (MO, P56, DH)

Participants indicated a need for standardised referral criteria and processes, as referrals were significantly influenced by the relationships between DHs and RH doctors, specific individuals and RH departments. Expectedly, staffing or resource limitations on weekends and public holidays had a low impact on non-emergency referrals but a higher impact on emergency referrals:

‘I can tell you if a patient will be accepted by knowing who is on call.’ (CM, P27, DH)

Other challenges included a lack of sufficient patient information, incomplete patient workups, particularly for emergency cases, and difficulties contacting DHs and arranging timely transport for patients being referred back to the DHs.

Access to timely and reliable transport services for both emergency and non-emergency referrals was perceived as particularly challenging, especially for non-emergency patients. Most participants (68%) from DHs reported the availability and timeliness of non-emergency transport as a major bottleneck, needing system-wide improvement. Non-emergency transport was not readily available and often required extensive pre-booking. This inadequacy was further reflected in the concerning proportion of respondents (DH 26%, RH 44%) who rated the overall quality of transport for non-emergencies as ‘poor’. While emergency transport was perceived to have its challenges, ambulances and personnel shortages were the biggest barriers (93%), followed by delays of 2–6 h (56%) for critically ill patients (triaged as red).

Beyond the mentioned challenges, responses revealed that these issues were often symptomatic of broader systemic barriers hindering timely access to care. This theme exposed a complex interaction between technological limitations, logistical obstacles and communication failures characterised by delays and inefficiencies that negatively impacted both patients and healthcare providers. Participants mainly from the DHs identified slow technology, particularly unreliable internet access, lack of real-time communication and reliance on slower communication channels such as email used for non-emergency referrals and patient tracking difficulties as common obstacles:

‘Delayed response, need to organise a follow-up booking date, clinics are overbooked.’ (CS, P62, DH)

‘The response is not as prompt as I would like it to be. By the time we receive a response from the consultant, the patient needs to be traced (majority of the patients do not have cell phone numbers) and then an additional appointment needs to be made to see the patient in the clinic on our side which takes up space for another patient that could likely have been seen.’ (MO, P18, DH)

Participants expressed their concerns about this heavy reliance on time-consuming methods such as the physical tracing of patients by community health care workers, extended waiting times for responses and appointment backlogs. This was further exacerbated by a lack of patient financial resources to pay for long-distance travel for their appointments, which was a significant barrier for patients because of the long waiting lists for non-emergency transport, which contributed to patients having to wait longer for care:

‘Availability of transport to the regional hospital, long waiting time for response and loss to follow-up. Not able to trace or contact patient to inform of appointment.’ (FP, P43, DH)

Participants described how these delays created a domino effect, affecting both patients and the healthcare system. Consequently, patients faced prolonged waiting times for responses, increased risk of lost-to-follow-up and increased frustrations. Simultaneously, healthcare providers had to manage overbooked clinics and strained resources within this complexity of referrals. One respondent eloquently captured this struggle, stating that:

‘Contacting the patient again and needing them to come back for further investigations or plans is troublesome for the patient and the health system.’ (FM Reg, P24, DH)

Participants also highlighted that resource scarcity at the RH potentially led to inappropriate gatekeeping practices that prioritised bed availability over timely patient care:

‘The excuse of “no bed space”. “It gives junior staff the power to not accept patients and should not be a factor AT ALL. Bed availability should have no influence on referral decisions. If no beds are available, then the hospital should have plans in place to make beds or escalate the problem. Patients should receive care at the appropriate level of hospital and junior clinicians should not be the ones making decisions based on bed availability.”’ (CM, P27, DH)

### Mismatched expectations and understanding

An overwhelming 88% of participants believed that improving RH doctors’ understanding and knowledge of how DHs work would enhance referral processes. This view was particularly strong among DH (97%) compared to RH participants (73%). This significant difference in perception (*p* = 0.001) highlighted a potential disconnect between the two and underscored a need for improved understanding between DH and RH doctors and potential knowledge-sharing initiatives to bridge the divide and optimise referral processes. District hospitals frequently perceived RH doctors as having unrealistic expectations of what can be done in some district facilities because of a lack of awareness about the limitations and restrictions at DHs. Participants believed that less frustration, more realistic expectations and smoother referrals could be achieved if RH doctors knew DHs’ limitations and restrictions:

‘Depending on the doctors on call at GRH – some Drs don’t understand the set-up at DH and have unrealistic expectations of what we can manage here and what we have access to.’ (CS, P47, DH)

‘Secondary institutions, especially extended departments be educated regarding limitations and restrictions in the periphery [*or*] rural areas. Understand unique challenges.’ (MO, P19, DH)

‘Some doctors do not understand circumstances and resource restraints at a DH and request further investigations which are only accessible during certain periods.’ (FP, P43, DH)

‘Lack of understanding on the part of the RH regarding resources at level one. For example, only 2 junior doctors on site after hours, unable to look after high care patients.’ (MO, P57, DH)

Mismatched expectations between the DHs and RH were also evident in perceptions of referral appropriateness. While 86% of DHs believed their referrals were appropriate, RH participants rated DH referrals as ‘fair’ for non-emergency (44%) and emergency (56%). Respondents emphasised that structured knowledge sharing and increased interaction could help bridge the gap between DHs and the RH. Participants highlighted the need for seniors to guide junior colleagues, ensuring quality interaction between DHs and RH:

‘The referrals from juniors are largely not good, referrals from seniors are appropriate, clear, and efficient. My feeling is that for the first month or two of new doctors working in a new hospital, all referrals should first be reviewed by senior before sending on to regional hospital whether in emergency or non-emergency situations.’ (SPC, P73, RH)

‘Often communicating with junior [*or*] inexperienced doctor[*s*] at RH.’ (FP, P23, DH)

### Building relationships

Participants emphasised the need for stronger relationships between DH and RH colleagues. They emphasised the need to increase face-to-face communications and interactions, regular meetings, introductions of new colleagues and feedback mechanisms. There was a preference for monthly or quarterly interaction, which could be achieved during in-reach, outreach or virtual sessions. There was a strong, shared belief among DH (92%) and RH (85%) doctors that a yearly meeting could improve inter-hospital relationships and communication:

‘It would be great if all departments could have a yearly meet & greet and explain their protocols [*or*] referral processes. I understand that this may not be feasible – a general email with their SOPs [*or*] invite to their Teams platform with access to clinical management protocols would also be helpful.’ (CS, P47, DH)

‘Meetings with different departments – with all the doctors: MO’s, registrars and consultants at least every 6 months.’ (Int, P13, DH)

‘Think it needs to be a quarterly meeting where people can present cases or address issues.’ (MO, P81, RH)

Participants believed that building familiarity and personal connections in professional settings among colleagues were crucial for effective communication and collaborative patient care:

‘More face-to-face interaction.’ (CS, P17, DH)

‘[*I*]t helps to know the people that you communicate with.’ (MO, P41, DH)

‘Visits to introduce yourself. Also give us some feedback on referred patients.’ (MO, P21, DH)

‘Introduction of new people employed. Even if it is electronically.’ (Spc, P79, RH)

‘A picture name hospital and rank of all doctors to be distributed amongst us. It’s good to see who you are speaking to.’ (HOCU, P82, RH)

**FIGURE 1:**
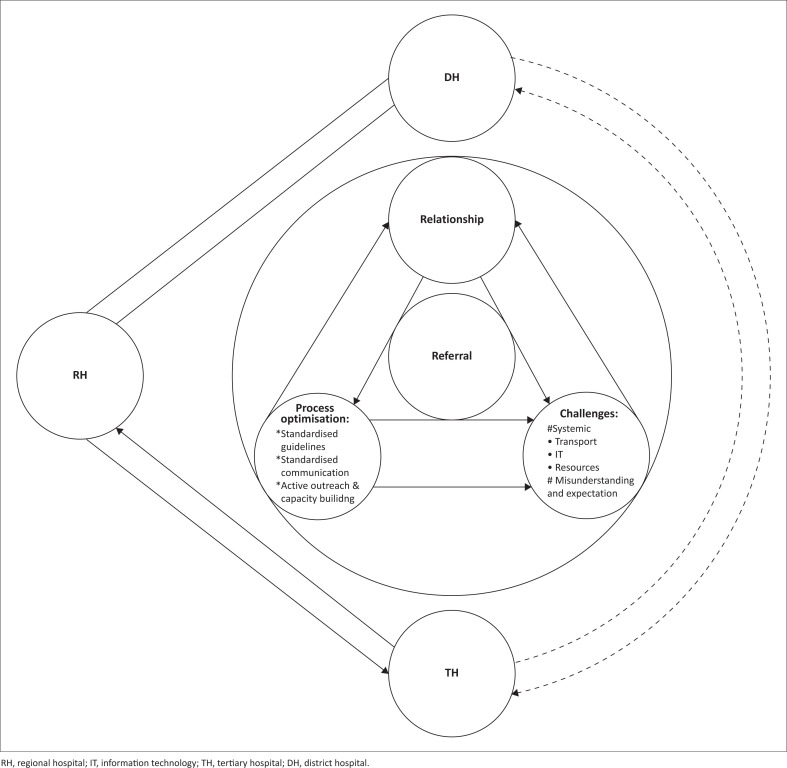
Referral pathways and the interplay between the main findings.

Figure 1 illustrates the interconnected relationships, processes and challenges of successful district-to-RH inter-referrals, creating a positive feedback loop of continuous improvement.

## Discussion

This study provided a comprehensive evaluation of patient referral processes for emergency and non-emergency conditions between DHs and an RH in two SA districts. To our knowledge, this study is the first to incorporate mixed methods to explore challenges at the primary–secondary care interface around referral processes in a rural South African setting. This multifaceted approach revealed that while there was general satisfaction with emergency patient referrals, non-emergency referrals presented significant difficulties. These challenges, broadly categorised as system-related or interpersonal, impacted various aspects of the referral pathway. System-level barriers included logistical issues, transport problems and a lack of standardised processes hindering timely access to care. Interpersonal challenges involved mismatched expectations and understanding between DHs and RH and strained communications and relationships. These challenges are most likely not unique to the study setting and are likely transferable to other resource-limited healthcare systems, where similar structural and operational barriers may exist. Effective, efficient and patient-centred referral processes are crucial for ensuring timely access to specialised care, optimising resource allocation within the healthcare systems, and ultimately improving patient outcomes.^27^ These processes are essential for ensuring a continuum of quality of care and saving lives.^4,28^

The unreliable and time-consuming nature of non-emergency transport caused by a lack of readily available transport options required long pre-bookings, resulting in significant referral delays. Previous studies also highlighted timely transportation as a significant barrier to an efficient referral.^29,30^

The complexity of referrals involved systemic issues relating to communication technology and referral guidelines. Non-emergency referrals via email were often perceived as slow and unreliable, with delayed responses. The Vula electronic app was not a standard mode of communication for emergency referrals, which led to some doctors responding to a referral made on Vula hours or days later. These delayed referral responses then led to difficulties in patient tracking and appointment backlogs, further exacerbated by the lack of reliable internet access in certain areas, which led to doctors using alternative modes of communication such as phone calls, WhatsApp and face-to-face communication. These findings were consistent with studies emphasising the importance of robust communication systems for effective referral processes.^31,32^ Furthermore, despite some participants being aware of referral guidelines, a lack of clarity, completeness and consistent accessibility hindered their effectiveness. Detailed, regularly updated, standardised and readily accessible referral guidelines would minimise ambiguity and ensure that all doctors had the same understanding of referral criteria and pathways.^33,34^

There was a preference for direct referral from the DHs to THs in some cases, bypassing the RH, as expressed by both DHs and the RH, with few advocating for this route for all patients in need of subspeciality care. While this study did not explore the reasons for this, some studies suggested that in some cases direct tertiary referrals could benefit patients with complex or time-sensitive problems that required prompt intervention because THs were better equipped with diagnostic and management capabilities.^35^ There appeared to be an apparent contradiction between the stated preference and the relatively low number of direct DH to TH referrals. Contributing factors could have included the perceived need for more RH guidance and involvement, resource limitations at THs, physician preferences and experiences and systemic barriers such as lack of established pathways or administrative procedures.^36,37^

There was a strong desire for improved communications and relationships between DHs and RH doctors. Most participants believed that regular meetings, knowledge-sharing initiatives and improved feedback mechanisms would enhance understanding, build trust and lead to smoother referral experiences. Notably, interpersonal professional relationships between DHs and RH doctors emerged as a crucial factor in facilitating effective referrals. This aligned with other studies that have highlighted the importance of communication and relationships in managing continuity between the primary and secondary care teams.^9^ Participants underlined the importance of fostering collaborative relationships through face-to-face or virtual meetings via online platforms to establish familiarity among colleagues, specifically for newly employed colleagues. These initial interactions were specifically designed to encourage informal interactions, joint training programmes and other opportunities for DHs and RH doctors to connect and strengthen interpersonal relationships.^9^

There were specific communication challenges within the referral process. While emergency referrals, primarily conducted via phone calls, were deemed satisfactory, non-emergency referrals, relying heavily on email communication, presented significant challenges. Participants advocated for an electronic referral tool with faster response times and standardised communication protocols across all DHs and RH departments. This aligned with existing research which demonstrated that structured referral tools could reduce delays and improve the quality of referral information.^17,30^

There was a lack of consistent feedback mechanisms within the referral system, with potentially missed opportunities for improvement of referrals, which aligned with a previous local study.^12^ Implementing standardised feedback mechanisms, potentially integrated into an electronic referral tool, could address this gap and enhance the effectiveness of the referral process. Investing in relationship-building initiatives and improving communication channels, including feedback pathways, could lead to tangible improvements in referral experiences for both healthcare providers and patients.^17,38^

### Strengths and limitations

Despite the recognised need for more research on patient referral processes,^34,39,40,41^ it remains largely understudied in South Africa. This study provided valuable insights into the referral processes between DHs and RHs in a rural district in SA. By sampling a diverse and representative doctors’ group, the study explored a comprehensive understanding of doctors’ perceptions, feelings, thoughts and lived experiences related to patient referrals.

However, there were certain limitations. While the study focused on the general referral process for both emergency and non-emergency cases, it did not focus on specific challenges inherent to referrals to particular departments. This broader approach, while valuable in identifying important system-level and interpersonal challenges, may not identify the nuances of specific clinical department pathways. The study also did not explore feedback practices and direct TH referrals in depth. These are areas for further research. The exclusion of transport personnel represented a missed opportunity to understand their perspective on transport-related delays, as one of the challenges identified. Lastly, the low survey response rate raised concerns about the potential for nonresponse bias. The perspectives of doctors who did not participate may differ systematically from those who completed the survey, which may limit the generalisability of the findings.

Despite these limitations, the study’s focus on a specific rural district in the WC province offers valuable insights that could apply to similar settings elsewhere.

## Conclusion

The aim of this study was to evaluate patient referral processes for non-emergency and emergency conditions in two districts of South Africa. This study highlighted the complexity of referrals leading to potential disconnections between DHs and the RH regarding the understanding of resources, capacity, limitations and services offered at each level. This mismatched understanding contributed to unrealistic expectations, referrals, delays in care and strained inter-facility relationships. Improving communication, building relationships and developing standardised referral guidelines and referral tools would potentially reduce these challenges.
